# Apolipoprotein E region molecular signatures of Alzheimer's disease

**DOI:** 10.1111/acel.12779

**Published:** 2018-05-23

**Authors:** Alexander M. Kulminski, Jian Huang, Jiayi Wang, Liang He, Yury Loika, Irina Culminskaya

**Affiliations:** ^1^ Biodemography of Aging Research Unit Social Science Research Institute Duke University Durham North Carolina

**Keywords:** age‐related phenotypes, aging, Alzheimer's disease, *APOE* polymorphism, health span, lifespan, neurodegenerative disorders

## Abstract

Although the *APOE* region is the strongest genetic risk factor for Alzheimer's diseases (ADs), its pathogenic role remains poorly understood. Elucidating genetic predisposition to ADs, a subset of age‐related diseases characteristic for postreproductive period, is hampered by the undefined role of evolution in establishing molecular mechanisms of such diseases. This uncertainty is inevitable source of natural‐selection–free genetic heterogeneity in predisposition to ADs. We performed first large‐scale analysis of linkage disequilibrium (LD) structures characterized by 30 polymorphisms from five genes in the *APOE* 19q13.3 region (*BCAM*,*NECTIN2*,*TOMM40*,*APOE*, and *APOC1*) in 2,673 AD‐affected and 16,246 unaffected individuals from five cohorts. Consistent with the undefined role of evolution in age‐related diseases, we found that these structures, being highly heterogeneous, are significantly different in subjects with and without ADs. The pattern of the difference represents molecular signature of AD comprised of single nucleotide polymorphisms (SNPs) from all five genes in the *APOE* region. Significant differences in LD in subjects with and without ADs indicate SNPs from different genes likely involved in AD pathogenesis. Significant and highly heterogeneous molecular signatures of ADs provide unprecedented insight into complex polygenetic predisposition to ADs in the *APOE* region. These findings are more consistent with a complex haplotype than with a single genetic variant origin of ADs in this region.

## 1 INTRODUCTION

Alzheimer disease (AD), a common form of dementia, is characterized by a progressive decline in cognitive function with age. AD affects 5.4 million Americans, and one of three elderly Americans will die with some form of AD‐type dementia. Genetic studies have identified a number of mutations in the *APP* gene (chromosome 21) and two homologous genes, *PSEN1* (chromosome 14) and *PSEN2* (chromosome 1), that appear to be causative in the early (familial) form of AD (Goate et al., 1991; Levy‐Lahad et al., 1995; Rogaev et al., 1995; Sherrington et al., 1995). Familial AD is much rarer than general ADs, which are prevalent after the age of about 65 years (Charlesworth, 1996). Although no genetic variants causative of late‐onset AD have been described to date, strong associations with this disease have been reported for genetic variants on chromosome 19 in the *APOE* gene region (19q13.3). The *APOE* e4 allele is associated with increased risk of ADs (Corder et al., 1993) and remains the most notable genetic risk factor for AD development in various populations (Raichlen & Alexander, 2014).

*APOE* encodes a protein involved in lipid homeostasis. In the brain, ApoE plays a role in astrocyte‐mediated amyloid‐beta degradation (Koistinaho et al., 2004), supporting the amyloid cascade hypothesis of AD development (Hardy & Higgins, 1992). However, some researchers contend that variants of other genes in the *APOE* region play a role in AD development. For example, the mitochondrial cascade hypothesis holds that *TOMM40* plays a role in AD development through the regulation of mitochondrial biogenesis (Roses et al., 2010; Swerdlow, Burns & Khan, 2014). *NECTIN2*, which flanks *TOMM40*, encodes a plasma membrane component of adherens junctions that also serves as an entry mediator for certain mutant strains of herpes simplex virus. The pathogen hypothesis thus holds that *NECTIN2* is a causative factor in the development of ADs (Harris & Harris, 2015; Martin et al., 2000).

Despite considerable progress in research into genetic predisposition to ADs, with the greatest advances involving *APOE* research, progress in the development of therapeutic interventions has been slow, with a success rate of only 0.4% in clinical trials conducted between 2002 and 2012 (Cummings, Morstorf & Zhong, 2014). The corresponding 99.6% failure rate indicates that the mechanisms underlying the development of ADs remain poorly understood. A fundamental difficulty in elucidating the genetics of AD and other complex age‐related diseases characteristic of postreproductive life is the undefined role of evolution in establishing the disease mechanisms (Nesse, Ganten, Gregory & Omenn, 2012). This problem is complicated by recent changes in human life expectancy (Oeppen & Vaupel, 2002) and the fitness landscape (Corella & Ordovas, 2014; Crespi, Stead & Elliot, 2010; Kulminski, 2013; Vijg & Suh, 2005). Evolution‐related factors are inevitable sources of genetic heterogeneity in determining predisposition to ADs. Heterogeneity in the strongest genetic risk factor for ADs, the *APOE* e4 allele, is evidenced by differences between geographic gradients in the frequency of the e4 allele among AD‐affected and general populations. Indeed, for Caucasians, the AD gradient ranges from 40.5% in Southern Europe to 61.3% in Northern Europe (Ward et al., 2012), whereas in the general population, the gradient is much wider, ranging from 10% to 15% in Southern Europe to 40%–50% in Northern Europe (Gerdes, 2003). This heterogeneity suggests that individuals carrying the e4 allele might not develop an AD. These observations are supported by genetic studies, which show that even homozygous e4 carriers might not develop an AD (Corder et al., 1993).

Here, we examined the complex molecular landscape of the *APOE* region, harboring five genes (*BCAM*,* NECTIN2*,* TOMM40*,* APOE*, and *APOC1*) and represented by 30 single nucleotide polymorphisms (SNPs) available from common genotyping arrays, by performing the first reported large‐scale analysis of linkage disequilibrium (LD) structures in five cohorts comprising 2,673 AD‐affected and 16,246 unaffected individuals. We also examined heterogeneity in cross talk between these genes, characterized by complexity of the LD structures. Consistent with the undefined role of evolution in establishing mechanisms of age‐related traits, our results show that the heterogeneous molecular landscape of the *APOE* region in AD‐affected individuals differs from that in unaffected individuals. As unprecedented insight into the human nature of ADs, this difference demonstrates that ADs are associated with highly heterogeneous molecular signatures spanning the entire region of all five genes, more consistent with a complex haplotype than with a single genetic variant origin of ADs.

## 2 RESULTS

### 2.1 Study overview

Data were obtained from the Late‐Onset Alzheimer Disease Family Study (LOADFS), Health and Retirement Study (HRS), Cardiovascular Health Study (CHS), and the Framingham Heart Study original (FHS) and offspring (FHSO) cohorts (Tables 1 and S1). The analyses focused on 30 SNPs in the Hardy–Weinberg equilibrium (HWE), *p*_HW_ > 10^−3^, that do not exhibit strong LD (*r* ^2^ < 0.8), representing the *BCAM*,* NECTIN2*,* TOMM40*,* APOE*, and *APOC1* genes in region 19q13.3 (Table S2). For cross‐platform comparisons, we used directly genotyped and imputed SNPs. Sensitivity analyses were performed using directly genotyped SNPs only. The primary analysis focused on the LD structure of the 19q13.3 region, as represented by the 30 selected SNPs, and on contrasting the LD patterns between AD‐affected and unaffected individuals of Caucasian ancestry, men and women combined. Affliction status (cases) was characterized as the presence of an AD, defined as a dementia of Alzheimer type (*n* = 2,673). Individuals without an AD (*n* = 16,246) were classified as noncases. As expected, cases were mainly from the LOADFS (designed as a case–control study), and they were typically from earlier birth cohorts (Table S1). Unless explicitly stated, the results of LD analyses are presented using a haplotype‐based method (details in Section 4).

**Table 1:** Basic demographic information for the genotyped participants in the selected studies

Factor	LOADFS	HRS	CHS	FHS	FHSO
*N*	3,715	7,226	4,326	631	3,021
AD cases (%)	1,850 (49.8%)	263 (3.6%)	252 (5.8%)	205 (32.5%)	103 (3.4%)
Men (%)	1,395 (37.6%)	3,129 (43.3%)	1,884 (43.6%)	210 (33.3%)	1,383 (45.8%)
Birth year, mean (*SD*)	1,928.5 (12.5)	1,934.2 (8.4)	1,914.1 (5.7)	1,911.8 (4.2)	1,935.8 (9.6)
Age at baseline, mean (*SD*)	73.5 (12.5)	60.6 (8.7)	72.8 (5.6)	35.8 (4.2)	34.7 (9.7)
Age at the end of follow‐up, mean (*SD*), years	77.3 (10.9)	79.1 (8.1)	83.5 (5.4)	91.4 (4.8)	72.2 (9.2)
Follow‐up through	2015^a^	2012	2002	2012	2012

AD denotes Alzheimer's disease and related dementias.

N denotes genotyped sample after excluding individuals with missingness for SNPs greater than 5% and missing information on AD.

*SD* denotes standard deviation.

LOADFS = the NIA Late‐Onset Alzheimer's disease Family Study; HRS = the Health and Retirement Study; CHS = the Cardiovascular Health Study; FHS = the Framingham Heart Study original cohort; FHSO = the FHS Offspring cohort.

Large proportion of AD cases in LOADFS is due to case–control design.

Large proportion of AD cases in FHS is due to older age of participants of this cohort at the end of follow‐up (mean age is 91.4 years) and larger proportion of women (66.7%) who are at higher risk of AD.

a Information on age at onset of AD in LOADFS was not known for all cases.

Consistent with previous studies (Deelen et al., 2011; Fortney et al., 2015), our association analyses showed that minor alleles of rs2075650 and rs157580 (*TOMM40*) were associated with higher and lower risk of AD development, respectively (Table S2). The effect directions were consistent in all studies; the effect sizes varied markedly, ranging from 0.380 (*p* = 0.014) in the FHS cohort to 1.45 (*p* = 1.65 × 10^−78^) in the LOADFS cohort for rs2075650 and from −0.052 (*p* = 0.612) in the FHS cohort to −0.705 (*p = *5.98 × 10^−23^) in the LOADFS cohort for rs157580 (Figure S1 and Table S2).

### 2.2 LD structure of the *APOE* region

**Figure 1:**
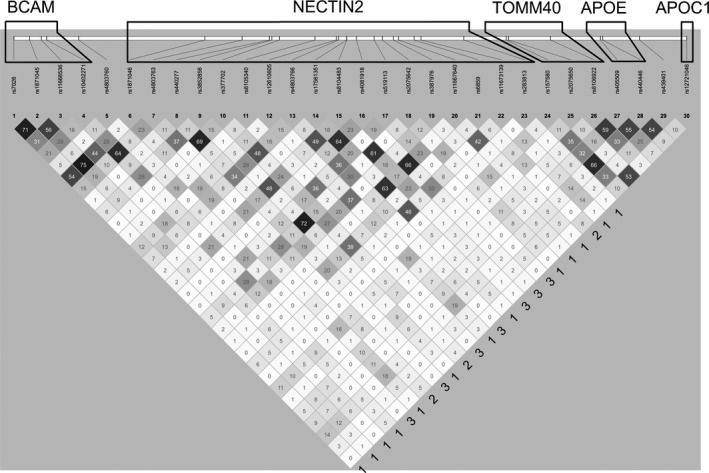
LD structure of the *APOE* region. LD (*r* ^2^, %) is shown in the pooled sample of all studies, cases and noncases combined, for 30 SNPs from the *BCAM*,*NECTIN2*,*TOMM40*,*APOE*, and *APOC1* genes. All *r* ^2^ > 0 were significant after conservative (because most SNP pairs could not be considered independent) Bonferroni correction, *p* < *p*_Bonf_ = 0.05/435 (= 30 × 29/2) = 1.2 × 10^−4^ (Table S3). Numbers 1–3 to the right show three patterns of LD between SNPs from the *TOMM40‐APOE‐APOC1* and *BCAM‐NECTIN2* loci. Pattern 1 is defined by a stronger LD for the *BCAM*‐*NECTIN2* SNPs with rs157580 than rs2075650. Pattern 2 is defined by about the same modest LD for the *BCAM*‐*NECTIN2* SNPs with rs2075650 and rs157580. Pattern 3 is defined by weak LD for the *BCAM* and *NECTIN2* SNPs with rs2075650 and rs157580. LD structure for each cohort separately is presented in Table S3. Functional annotation of the 30 selected SNPs is given in Table S5A–C

We first examined the LD structure of the selected region in the large pooled sample of all cohorts (cases and noncases combined). It was represented by three heterogeneous clusters mapped to the *BCAM* and *NECTIN2* genes and the *TOMM40*‐*APOE*‐*APOC1* locus (Figure 1). Each cohort exhibited the same structure (Table S3). Stronger LD was observed for SNPs within each gene cluster. SNPs from the *BCAM*‐*NECTIN2* locus were in low‐to‐moderate LD with SNPs from the *TOMM40‐APOE‐APOC1* locus.

### 2.3 Molecular signatures of ADs

Then, we evaluated the LD structure for SNPs in the *APOE* region for cases and noncases separately and contrasted LD patterns between these groups using haplotype‐ and genotype‐based methods. We used these two methods because differences in the LD estimates from them are informative of deviation from HWE. This information is important because HWE in the entire sample does not guarantee HWE in subsamples, and thus, the observed deviation from HWE may be biologically plausible. Although such deviation can be readily identified by estimating HWE in subsamples separately (e.g., *p*_HW_ = 5.94 × 10^−7^ in cases, whereas *p*_HW_ = 0.665 in noncases for rs11668536; Table S2), it can also occur regardless of HWE in subsamples at the haplotype level, that is, when Δ_AB_ ≠ *D*_AB_, which is difficult to detect in stratification analyses (see Section 4).

**Figure 2:**
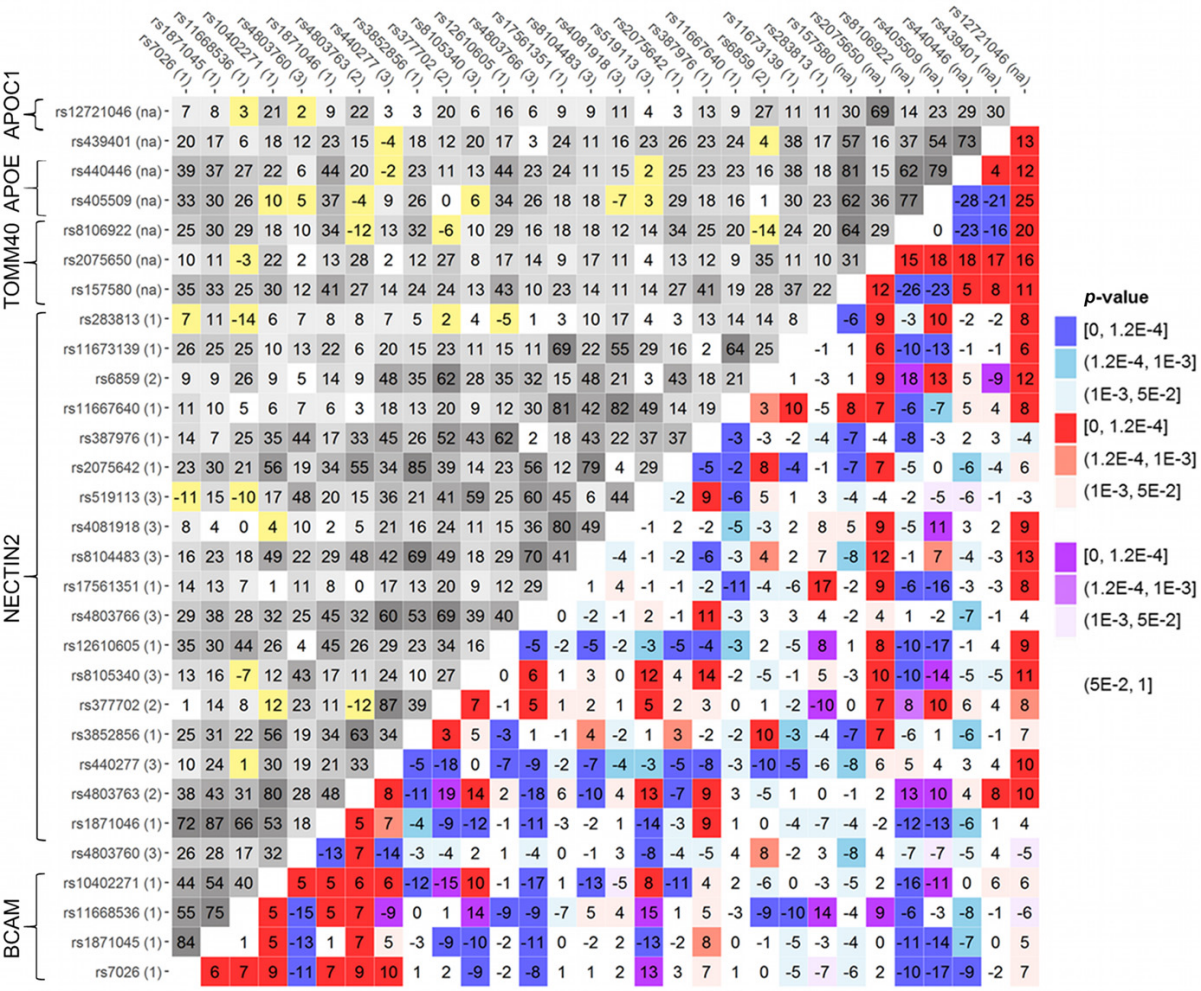
LD structure in AD‐unaffected individuals and the difference in LD in subjects with and without ADs. Upper‐left triangle: LD pattern (*r*, %) in the pooled sample of all studies, noncases, for 30 SNPs. Lower‐right triangle: heat map for Δ*r* representing the molecular signature of ADs as the difference in LD in subjects with and without AD. The difference Δ*r* was defined as Δ*r* = *r*_cases_–*r*_noncases_ if LD coefficients *r* were of opposite signs in cases and noncases (yellow and purple); otherwise, Δ*r* was defined as Δ*r* = |*r*_cases_|–|*r*_noncases_|. Red denotes *r*_cases_ > *r*_noncases_, and blue denotes *r*_cases_ < *r*_noncases_. Numbers 1–3 after SNP IDs indicate patterns shown in Figure 1. Legend on the right shows color‐coded *p*‐values of difference Δ*r*. We used *r* rather than *r* ^2^ here to emphasize that *r* can be of opposite sign in cases and noncases. The heat map shows that LD in cases changes for vast majority of SNPs in the entire region spanning all five genes. Figure S3 shows the heat map for *r* ^2^. Numerical estimates are shown in Table S3

We found that the LD patterns estimated using the haplotype‐based method differed significantly between cases and noncases in the pooled sample of all cohorts (*p *<* *2 × 10^−4^) and each of the four cohorts: LOADFS (*p *<* *2 × 10^−4^), HRS (*p = *1.8 × 10^−2^), CHS (*p = *1.4 × 10^−3^), and FHSO (*p = *1.5 × 10^−2^). In the FHS cohort, the difference was not significant (*p = *0.908). The patterns of the differences represent molecular signatures of ADs in this genetic region. The molecular signatures in the pooled sample are illustrated by heat maps for Δ*r* (Figure 2) and Δ*r* ^2^ (Figure S3).

Our analysis identified 173 of 435(= 30 × 29/2) SNP pairs (39.8%) with Δ*r* values significant at the Bonferroni‐adjusted level: *p *≤ *p*_Bonf_ = 1.2 × 10^−4^. For 27 additional SNP pairs, we observed suggestive significances: *p*_Bonf_ < *p *< 10^−3^. Of these 200 SNP pairs (46.0%), the correlation coefficients *r* for 17 SNP pairs with significant Δ*r* (*p *≤ *p*_Bonf_) were in opposite directions for AD cases and noncases. Such significant differences could be missed when using *r* ^2^ statistics (Figure S3). Figure 2 illustrates the complex rearrangement of LD in cases compared with noncases spanning the entire region.

Molecular signatures of ADs estimated using the genotype‐based method (Figure S4 and Table S4) were qualitatively the same as those estimated using the haplotype‐based method, with significant differences observed between cases and noncases in the pooled sample of all cohorts (*p *<* *2 × 10^−4^) and in each of the four cohorts: LOADFS (*p *<* *2 × 10^−4^), HRS (*p = *0.022), CHS (*p = *0.014), and FHSO (*p = *0.024), but not in the FHS (*p = *0.926). The genotype‐based method provided 140 SNP pairs significant at *p* < *p*_Bonf_, of which 135 SNP pairs attained *p *< *p*_Bonf_, and five SNP pairs were of suggestive significance (*p*_Bonf_ < *p *< 10^−3^) according to the haplotype‐based method. For 28 additional SNP pairs, we observed suggestive significance (*p*_Bonf_ < *p *< 10^−3^) according to the genotype‐based method.

**Figure 3:**
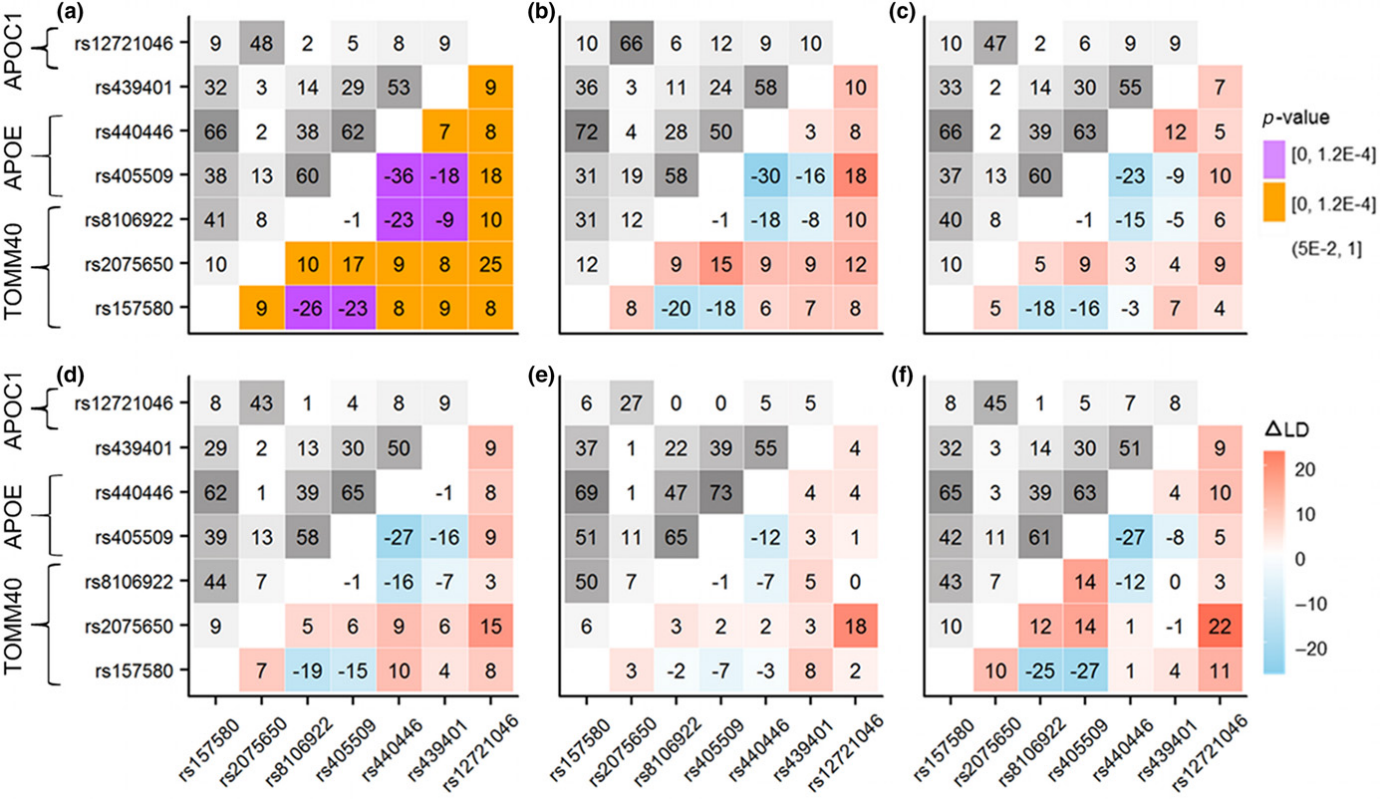
Molecular signature of ADs in the *TOMM40‐APOE‐APOC1* locus. (a) Pooled sample of all cohorts, (b) Late‐Onset Alzheimer Disease Family Study, (c) Health and Retirement Study, (d) Cardiovascular Health Study, (e) Framingham Heart Study (FHS) original cohort, and (f) FHS offspring cohort. Upper‐left triangle: LD pattern (*r* ^2^, %) in noncases for seven SNPs representing the *TOMM40‐APOE‐APOC1* locus. Lower‐right triangle: heat maps for $\Delta r^{2}=r_{\mathrm{cases}}^{2}-r_{\mathrm{noncases}}^{2}$ representing the molecular signature of ADs in this locus. Color in (a) codes *p*‐values; color in (b–f) codes Δ*r* ^2^ (see legend). Numerical estimates are shown in Table S3. See Figure S5 for heat maps for directly genotyped SNPs

Notably, Figure 3 shows that the molecular signatures of ADs for seven SNPs in the *TOMM40‐APOE‐APOC1* locus were mostly consistent in all cohorts. Accordingly, all pairwise estimates of Δ*r* in the pooled sample (except the rs8106922 [*TOMM40*] and rs405509 [*APOE*] pair) were significant at *p *< *p*_Bonf_. Figure 3a shows that the molecular signature of AD in this locus was associated with increasing LD for some SNP pairs and decreasing LD for others, in cases as compared with noncases. For example, LD of the rs2075650 SNP (*TOMM40*), with the minor allele exhibited a strong detrimental association with ADs (Table S2), increased with all of the other six SNPs in this locus, whereas LD of rs157580 (protective association) increased with rs2075650, rs440446, rs439401, and rs12721046 and decreased with rs8106922 (*TOMM40*) and rs405509 (*APOE*). As shown in Figure S5, the observed patterns in the *TOMM40‐APOE‐APOC1* locus were not altered by imputation.

### 2.4 Heterogeneous connections between the *BCAM‐NECTIN2* and *TOMM40‐APOE‐APOC1* loci in AD‐affected and unaffected individuals

**Figure 4:**
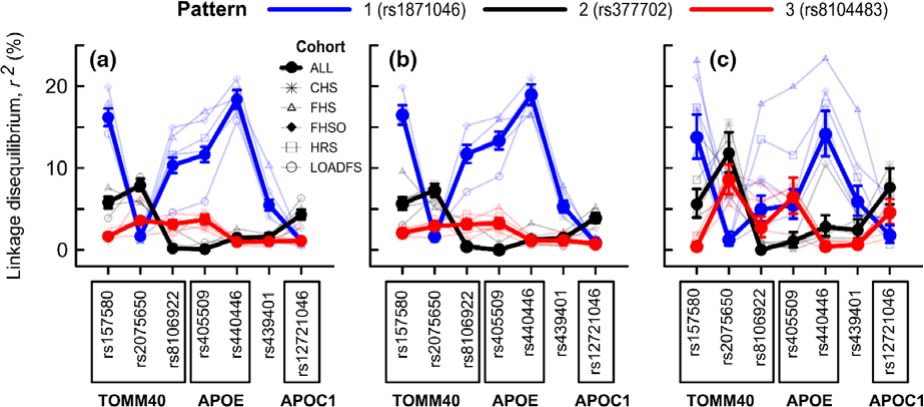
LD between selected SNPs from the *BCAM*‐*NECTIN2* and *TOMM40‐APOE‐APOC1* loci. SNPs rs1871046, rs377702, and rs8104483 are representative of patterns 1 (light/dark blue), 2 (gray/black), and 3 (light/dark red), respectively, as shown in Figure 1. Symbols denote samples. The 95% confidence intervals are shown in the pooled sample of all cohorts (“All”). (a) A sample of cases and noncases combined. (b) Noncases only. (c) Cases only. LD for the other SNPs from these patterns in the pooled sample of all cohorts is illustrated in Figure S2

Figure 1 highlighted heterogeneous structure of LD in the *APOE* region, which was dominated by the LD structure in noncases (Table S3 and Figure S3) due to their sixfold excess (16,246 noncases vs. 2,673 cases). Visual analysis of Figure 1 indicates two heterogeneous structures connecting the *BCAM‐NECTIN2* and *TOMM40‐APOE‐APOC1* loci. One structure was evidenced by three types of ad hoc LD patterns between multiple SNPs from the *BCAM‐NECTIN2* and *TOMM40‐APOE‐APOC1* loci indicated in Figure 1 and further illustrated in Figures 4 and S2. The other structure is observed within these ad hoc LD patterns, as they held for the *BCAM* and *NECTIN2* SNPs regardless of LD. For example, pattern 1 was the same for rs12610605 and rs11673139 (all *NECTIN2*), despite the low LD between these SNPs (*r* ^2^ = 2%). The same pattern was observed for SNPs #12 and #19, which exhibited higher LD (*r* ^2^ = 37%) and for SNPs #9 and #18, which exhibited even higher LD (*r* ^2^ = 72%) (Figure 1). Pattern 2 also held regardless of LD between the *BCAM* and *NECTIN2* SNPs (e.g., LD between SNPs #7 and #10 was <1%). Despite the apparently modest LD for SNPs between the *BCAM*‐*NECTIN2* and *TOMM40‐APOE‐APOC1* loci (*r* ^2^ < 20%), these patterns were consistent in all cohorts of cases and noncases combined and noncases only (Figure 4a,b).

As shown in Figure 2, there were significant changes in LD between cases and noncases for 23 SNPs from the *BCAM‐NECTIN2* locus with 7 SNPs from the *TOMM40‐APOE‐APOC1* locus. This rearrangement of the LD structure in cases compared with noncases is indicative of AD‐specific cross talk between these genes. Figure 4 shows that these three ad hoc LD patterns in cases substantially differ from those in noncases. Visual analysis of Figures 2 and S3 also suggests that changes in LD between cases and noncases differ between these ad hoc LD patterns. To quantify this visual insight, we analyzed the correlation (Pearson two‐tailed test) in the change in LD magnitude between cases and noncases (characterized by $\Delta r^{2}=r_{\mathrm{cases}}^{2}-r_{\mathrm{noncases}}^{2}$) with the LD in noncases ($r_{\mathrm{noncases}}^{2}$) for SNP pairs comprised of the intersection of the 23 SNPs from the *BCAM*‐*NECTIN2* locus and seven SNPs from the *TOMM40‐APOE‐APOC1* locus. In this analysis, we used 58 SNP pairs for which Δ*r* was significant at *p *< *p*_Bonf_ = 1.2 × 10^−4^ (Figure 2). A significant inverse correlation was observed between *Δr* ^2^ and $r_{\mathrm{noncases}}^{2}$ for these pairs (*r*_Pearson_ = −0.58, *p = *2.0 × 10^−6^), which was driven by SNPs from pattern 1 (*r*_Pearson_ = −0.82, *p* = 6.8 × 10^−10^) (*n*_S_ = 37). For SNPs from patterns 2 and 3, we observed a significant direct correlation (*r*_Pearson_ = 0.72, *p = *2.1 × 10^−4^) (*n*_S_ = 21) that was consistent for each pattern (i.e., *r*_Pearson_ = 0.81, *p = *2.4 × 10^−3^ [*n*_S_ = 11] for pattern 2 and *r*_Pearson_ = 0.64, *p* = 4.4 × 10^−2^ [*n*_S_ = 10] for pattern 3).

### 2.5 LD and minor allele frequency (MAF)

**Figure 5:**
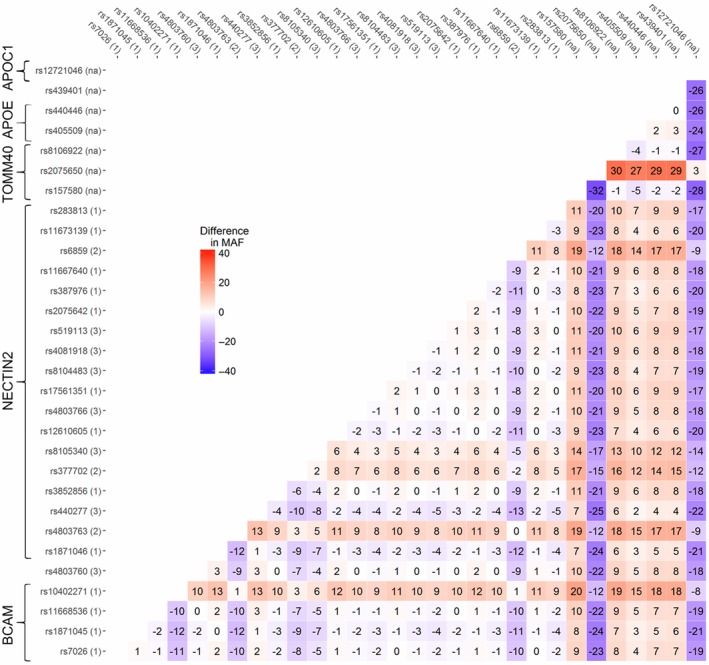
Heat map for the difference of MAF between AD cases and noncases. The difference was defined as ΔMAF = (${\text{SNP}}_{\mathrm{cases}}^{1}-{\text{SNP}}_{\mathrm{noncases}}^{1}$) – (${\text{SNP}}_{\mathrm{cases}}^{2}-{\text{SNP}}_{\mathrm{noncases}}^{2}$). Numbers 1–3 after SNP IDs indicate patterns shown in Figure 1. Legend in the inset shows color‐coded difference in MAF

We also examined whether differences in MAF between cases and noncases create the molecular signatures of ADs (Figure 2), even though the complex heterogeneous structure of the AD molecular signatures suggested that this is unlikely. To examine this question quantitatively, we compared the correlation between Δ*r* and change in the differences in MAF between cases and noncases (i.e., ΔMAF = $\left[{\text{SNP}}_{\mathrm{cases}}^{1}-{\text{SNP}}_{\mathrm{noncases}}^{1}\right]$ – $\left[{\text{SNP}}_{\mathrm{cases}}^{2}-{\text{SNP}}_{\mathrm{noncases}}^{2}\right]$) for the same SNP pairs. There was no significant correlation for the SNP pairs over either the entire region (*r*_Pearson_ = −0.017, *p = *0.725) or the *TOMM40*‐*APOE‐APOC1* locus (*r*_Pearson_ = −0.103, *p = *0.655). This result provided quantitative support for the apparent difference in structures of the heat maps for ΔMAF (Figure 5) and Δ*r* (Figure 2).

### 2.6 Regulatory features and expression quantitative trait loci (eQTL) evidence

Using the variant effect predictor from Ensembl (http://www.ensembl.org) (McLaren et al., 2010), nine of the 30 SNPs were annotated as functional variants in the promoter or promoter flanking regions that regulate the transcriptional activity of the *BCAM*,* NECTIN2*,* TOMM40*,* APOE*, and *APOC1* genes (Table S5A). Another study also reported that rs405509 regulates *APOE* promoter activity (Artiga et al., 1998). Rs387976 (*NECTIN2*), located in an open chromatin region, seems to mediate transcription factor binding (Song et al., 2011). Ten functional variants were found to exist in active expression states in multiple cell lines, ranging from 1 (rs387976) to 63 (rs157580 [*TOMM40*]) of 68 cell types with data available from Ensembl. All of these variants can be in a poised state in one or more cell types (i.e., they can be epigenetically activated at a later stage in development or in response to exogenous stimuli) (Creyghton et al., 2010; Murao, Noguchi & Nakashima, 2016; Puri, Gala, Mishra & Dhawan, 2015). Two variants, rs1871046 (*NECTIN2*) and rs440446 (*APOE*), were characterized as being a poised expression state in many cell types (20 and 32 epigenomes, respectively). In addition, seven of 10 regulatory variants exhibited a poised epigenetic signature in normal human astrocytes (NHAs), and one of the variants (rs439401) was found to be active in these cells.

Functional annotation using HaploReg (Ward & Kellis, 2012) identified two evolutionarily conserved variants (rs7026 [*BCAM*], rs6859 [*NECTIN2*]), 11 variants in promoter histone marks, 26 variants in enhancer histone marks, and 25 variants in DNase clusters assayed in different tissue types (Table S5B). The analysis showed that 26 variants affect regulatory motifs, 11 variants bind regulatory protein, and most of the SNPs were identified as eQTL for the five genes in the *APOE* region in at least one cell type. Variants rs387976, rs12610605, and rs8105340 (all *NECTIN2*) were identified as possible eQTL for the *RELB*,* GEMIN7*, and *PVR* genes, respectively, in specific cell types. Six variants (rs1871046, rs157580, rs2075650, rs405509, rs440446, and rs439401) were found to have multiple regulatory features (from 7 to 23) in a variety of tissues.

Most SNPs were also identified as eQTL using GTEx (Consortium 2015) for the same protein‐coding genes in which they are located (Table S5C). Variant rs11667640 (*NECTIN2*) was also predicted to be an eQTL for the nearby *APOC2* gene. Variant rs283813 (*NECTIN2*) was predicted as an eQTL for the nearby *BCAM* gene, which is expressed in skin, and for *ZNF155*, which is expressed in the putamen region of the brain. The *ZNF155* gene maps to a zinc finger gene cluster located on 19q13. Highly significant expression of all of these protein‐coding genes was detected in multiple tissues.

## 3 DISCUSSION

Our results provide compelling evidence that AD in humans in the *APOE* region is associated with a highly heterogeneous molecular signature represented by the pattern of the differences in LD structures between AD‐affected and unaffected individuals (Figure 2). This signature includes SNPs from all five genes (i.e., it spans the entire region). Remarkably consistent AD signatures were observed in the locus consisting of the *TOMM40*,* APOE*, and *APOC1* genes (Figure 3). LD rearrangements between SNPs in the entire region consisted of a mixture of same‐ and opposite‐direction changes in LD for specific SNP pairs, likely driven by the heterogeneous structure of connections between SNPs from different genes, as discussed below. Significant changes of LD highlighted by AD signatures indicate SNPs, which are likely involved in AD pathogenesis.

We elucidated the heterogeneous structure of connections of SNPs from the *BCAM‐NECTIN2* locus with SNPs from the *TOMM40‐APOE‐APOC1* locus. The heterogeneity is evidenced by: (i) three qualitatively different patterns of LD for SNPs from these two loci (Figure 4) and (ii) the same LD structure in each pattern for different SNPs from the *BCAM‐NECTIN2* locus with SNPs from the *TOMM40‐APOE‐APOC1* locus, regardless of LD for SNPs in the *BCAM‐NECTIN2* locus (Figure 1). This heterogeneity indicates complex cross talk between genes from these two loci supported by different alleles from these genes in different groups of individuals. Our findings indicate that LD in these three patterns of heterogeneous connections between the *BCAM‐NECTIN2* and *TOMM40‐APOE‐APOC1* loci substantially changes in AD‐affected individuals compared with unaffected individuals (Figure 4). Specifically, for pattern 1, LD between SNPs from the *BCAM*‐*NECTIN2* and *TOMM40‐APOE‐APOC1* loci is lower in AD‐affected individuals compared with unaffected individuals. Notably, this difference indicates weakening of LD between SNPs from the *BCAM‐NECTIN2* locus and the rs157580 and rs8106922 SNPs from the *TOMM40* gene, in which the minor alleles are correlated with lower AD risk. For patterns 2 and 3, we observed increases in LD between SNPs from the *BCAM*‐*NECTIN2* locus and SNPs from the *TOMM40‐APOE‐APOC1* locus in AD‐affected individuals. For pattern 2, this change results in a strengthening of LD for the rs2075650 (*TOMM40*) and rs12721046 (*APOC1*) SNPs with minor alleles correlated with higher AD risk. For pattern 3, this result suggests that minor LD between SNPs from these two loci in unaffected individuals becomes stronger in AD‐affected individuals.

Our results show that the molecular signatures of ADs cannot be explained by differences in the MAF for AD‐affected and unaffected individuals. Accordingly, the molecular signatures of ADs are consistent with the cis‐(haplotype) rather than a single allele origin of ADs (Jazwinski et al., 2010; Lescai et al., 2011). Whether the molecular signatures of ADs include the *APOE,* e4 allele remains unclear. However, because rs2075650 in Caucasians is typically in modest LD with rs429358 (*r* ^2^~0.5), which defines the *APOE* e4 allele, the signature likely includes the e4 allele. Assuming an evolutionary origin of LD structures in unaffected individuals, these results are consistent with the uniquely human nature of ADs, which are sensitive to the modern environment (Finch, 2012).

Finally, the results of our bioinformatics analysis show that 10 of 30 SNPs in the *APOE* region are regulatory variants in active expression states in a variety of tissues in from 1 to 63 of the 68 cell types available for analysis. Variant rs439401 in the *APOE‐APOC1* intergenic region, which includes a specific astrocyte enhancer for the *APOE* gene (Grehan, Tse & Taylor, 2001), is the only variant among the 10 active expression SNPs that is active in NHAs. However, seven other variants exhibit a poised epigenetic signature in NHAs. Astrocytes have important functions in brain development, physiology, and health. They also serve as neural stem cells in the adult brain and have been implicated in various pathologic processes, including ADs (Pekny et al., 2016). Recent data indicate that the function of astrocytes can change from potent pro‐inflammatory to potent anti‐inflammatory in response to regulatory signals (Sofroniew, 2015). Genes in poised expression states in relevant cell types can be activated by changes in the epigenome later in development or by environmental cues (Creyghton et al., 2010; Murao et al., 2016; Puri et al., 2015). These observations strongly suggest that AD development could be the result of a complex transcriptional regulatory structure modulating regional gene expression (Fitzsimons et al., 2014) supported by clustering of alleles in the molecular signatures identified in the *APOE* region. Given the functional role of *BCAM*,* NECTIN2*,* TOMM40*,* APOE*, and *APOC1* genes and the regulatory activity of variants in these genes, the molecular signatures elucidated in the present study could be associated with increased risk of developing an AD by increasing susceptibility to brain infections (Itzhaki et al., 2016; Porcellini, Carbone, Ianni & Licastro, 2010).

Despite the rigor of this study and reliability of our results, there are potential limitations. First, the available data did not allow us to investigate the role of the *APOE* e2 and e4 alleles in the identified molecular signatures of ADs. Second, we did not compare the molecular signatures of ADs in males and females because of the limited sample size. Third, despite validation of our findings in four independent studies, further replication in larger samples would improve the characterization of the molecular signatures of ADs.

In conclusion, significant and highly heterogeneous molecular signatures of ADs provide unprecedented insight into complex polygenetic predisposition to ADs in the *APOE* region. These findings are more consistent with a complex haplotype than with a single genetic variant origin of ADs in this region.

## 4 EXPERIMENTAL PROCEDURES

### 4.1 Data availability

This manuscript was prepared using a limited access datasets obtained through dbGaP and the University of Michigan. Phenotypic HRS data are available publicly and through restricted access from http://hrsonline.isr.umich.edu/index.php?p=data.

### 4.2 Accession numbers

This manuscript was prepared using a limited access datasets obtained through dbGaP (accession numbers phs000007.v28.p10, phs000287.v5.p1, phs000428.v1.p1, and phs000168.v2.p2) and the University of Michigan. Phenotypic HRS data are available publicly and through restricted access from http://hrsonline.isr.umich.edu/index.php?p=data. See also Text S1.

### 4.3 Experimental design

We used data from five cohorts (described below) to examine linkage disequilibrium (LD) structure of the *APOE* region spanning five genes (*BCAM*,* NECTIN2*,* TOMM40*,* APOE*, and *APOC1*), using 30 single nucleotide polymorphisms (SNPs). The selected SNPs did not exhibit strong LD (*r* ^2^ < 0.8) and were directly genotyped in at least two cohorts. We focused on analysis of the LD structure in the entire sample of individuals of Caucasian ancestry (men and women combined) and on comparative analyses of the LD structures in individuals affected and unaffected by Alzheimer's disease (AD).

### 4.4 Study cohorts and phenotypes

Data were drawn from the Framingham Heart Study original (FHS) and offspring (FHSO) cohorts (Cupples, Heard‐Costa, Lee & Atwood, 2009), the Cardiovascular Health Study (CHS) (Fried et al., 1991), the Health and Retirement Study (HRS) (Juster & Suzman, 1995), and the NIA Late‐Onset Alzheimer Disease Family Study (LOADFS) (Lee, Cheng, Graff‐Radford, Foroud & Mayeux, 2008) for individuals who identified themselves as of Caucasian ancestry. The LOADFS was designed to ascertain dementias of Alzheimer type in the elderly and recruited cases and controls. The FHS and CHS collected information on ADs in population samples during follow‐up. In LOADFS, FHS, and CHS, AD was defined based on diagnoses made according to National Institute of Neurological and Communicative Disorders and Stroke and Alzheimer's disease and Related Disorders Association. The HRS is linked with Medicare service use files and includes enrollment information as well as the diagnoses made (International Classification of Disease [ICD]‐revision 9, Clinical Modification) during episodes of care paid for by the Medicare system. A diagnosis of AD was defined based on ICD‐9:331.0× in claims paid for by either Medicare Part A (facility‐based services [e.g., hospitals]) or Medicare Part B (professional services [e.g., physician practices]). Our analyses included 1,850 cases from the LOADFS, 263 cases from the HRS, 252 cases from the CHS, 205 cases from the FHS, and 103 cases from the FHSO. Individuals with no AD constituted the noncase group: *n = *1,865 in the LOADFS, *n* = 6,963 in the HRS, *n* = 4,074 in the CHS, *n* = 426 in the FHS, and *n* = 2,918 in the FHSO. Basic demographic information for the participants of each cohort is given in Tables 1 and S1. As this study was not intended to separate familial and nonfamilial cases of AD, we used both FHS and FHSO cohorts. Note that familial component in the cohorts of the FHS and FHSO survivors is small because of small sample of genotyped FHS survivors (*n* = 631) compared with the FHSO survivors (*n* = 3,021).

### 4.5 Genotypes

Genotyping was performed using the same customized Illumina iSelect array (the IBC‐chip, ~50K single nucleotide polymorphisms [SNPs]) in the FHS and CHS cohorts, Affymetrix 500K in the FHS, Illumina HumanCNV370v1 chip (370K SNPs) in the CHS, Illumina HumanOmni 2.5 Quad chip (~2.5 M SNPs) in the HRS, and Illumina Human 610Quadv1_B Beadchip (~610K SNPs) in the LOADFS.

The analyses focused on 30 SNPs representing the *BCAM‐NECTIN2‐TOMM40‐APOE‐APOC1* locus in the 19q13.3 region (Table S2). These SNPs were selected because they were not in strong linkage disequilibrium (LD), with *r* ^2^ < 0.8, and were directly genotyped in at least two cohorts. We excluded individuals with >5% missingness. To facilitate cross‐platform comparisons, we selected directly genotyped target SNPs or their proxies (*r* ^2^ > 0.8 in the 1,000 Genomes Project, CEU population) using all available arrays for each study. Nongenotyped SNPs were imputed (IMPUTE2, (Howie, Donnelly & Marchini, 2009)) according to the 1,000 Genomes Project Phase I integrated variant set release (SHAPEIT2) in the NCBI build 37 (hg19) coordinate. Only SNPs with high imputation quality (info>0.8) were retained for the analyses, which resulted in the exclusion of four SNPs from the FHS/FHSO (rs11668536, rs440277, rs4803760, and rs7026), with info<0.66 (details in Table S2).

### 4.6 Association analysis

Associations between ADs and each of the 30 selected SNPs were evaluated using an additive genetic model with the minor allele as an effect allele. Given limited information on AD age at onset in the LOADFS, the associations in this study were characterized using a logistic model with AD as a binary outcome and random effects to adjust for potential familial clustering (*gee* package in r). Associations in the other studies were evaluated using the Cox proportional hazard mixed‐effects regression model (*coxme* package in r) to adjust for familial clustering. The time variable in the Cox model was the age at onset of AD or the age at right censoring in 2002 for the CHS, 2012 for the FHS, and 2012 for the HRS. All statistical tests were adjusted for: (all studies) age, sex; (CHS) field center; (FHS) whether the DNA samples had been subject to whole‐genome amplification, (HRS) HRS cohorts. Meta‐statistics were evaluated using METAL (Willer, Li & Abecasis, 2010).

### 4.7 LD analysis

LD was characterized by the correlation coefficient *r* using haplotype‐based (Weir, 1979) and genotype‐based (Zaykin, Meng & Ehm, 2006) methods. Specifically, the haplotype‐based method evaluates *r* as

$$r_{A,B}^{h}\;=\;D_{\mathrm{AB}}/\sqrt{p_{A}\left(1-p_{A}\right)p_{B}\left(1-p_{B}\right)},$$

where *p*_*i*_ (*i* = *A,B*) are allele frequencies in two SNPs, *D*_*AB*_ = *h*_*1*_ *– p*_*A*_ *p*_*B*_ is the LD coefficient, and *h*_*1*_ is the frequency of a haplotype *AB*. This method assumes Hardy–Weinberg equilibrium (HWE), which may or may not hold in subsamples and/or at the haplotypic level, even when SNPs in a sample are in HWE (Nielsen, Ehm & Weir, 1998). Haplotype frequencies were evaluated using an expectation‐maximization algorithm (*haplo.stats* package in r).

The genotype‐based method evaluates *r* without assuming HWE, as

$$r_{A,B}^{g}\;=\;\Delta_{\mathrm{AB}}/\sqrt{\left(p_{A}\left(1-p_{A}\right)+\;D_{A}\right)\;\left(p_{B}\left(1-p_{B}\right)\;+\;D_{B}\right)},$$

where Δ_*AB*_ is the composite LD coefficient, defined as $\Delta_{\mathrm{AB}}=h_{1}+h_{1}^{\prime }-2p_{A}p_{B}$, and $h_{1}^{\prime }$ is the joint frequency of alleles *A* and *B* at two different gametes (Weir & Cockerham, 1979). *D*_A_ and *D*_B_ are HW disequilibrium coefficients at these SNPs. In the case of HWE, $h_{1}^{\prime }=p_{A}p_{B}$ implies that $r_{A,B}^{g}=r_{A,B}^{h}$, so Δ_*AB*_ is an unbiased estimate of the LD parameter *D*_*AB*_. Therefore, inequality Δ_*AB*_ ≠ *D*_*AB*_ characterizes deviation from the HWE at the haplotypic level, which otherwise could be difficult to detect (Nielsen et al., 1998).

Significance of the *r* ^2^ estimates was characterized using chi‐square statistics, defined as χ^2^ = *r* ^2^N, where *N = *2*n* is the number of gametes and *n* is the sample size (Lewontin, 1988). Given potential loss of power due to inferring haplotypes from genotypes (Wellek & Ziegler, 2009), we used a more conservative estimate, with *n* instead of *N*.

We employed a LD contrast test (Zaykin et al., 2006) to compare the LD patterns between the AD‐affected and unaffected groups. Given a set of *K* SNPs, we adopted the *Z*_2_ statistic, *Z*_2_ = trace ((*r*_1_ – *r*_0_)*T* (*r*_1_ – *r*_0_)), where *r*_1_ and *r*_0_ are the matrices of the LD correlation coefficients for AD‐affected and unaffected individuals, respectively. This statistic was used to characterize the significance of the overall difference in LD patterns between these two groups and the differences in pairwise estimates of LD between the groups. In the latter case, considering a pair of SNPs, *r*_1_ and *r*_0_ are simplified into a 2 × 2 matrix with two off‐diagonal coefficients representing the LD coefficient. Then, we have *Z*_2_ = 2(*r*_1_ – *r*_0_)^2^. To contrast LD between the AD‐affected and unaffected groups, we used a permutation procedure by shuffling the case and noncase labels (Krzanowski, 1993) to obtain an empirical distribution of *Z*_2_ under the null hypothesis *r*_1_ = *r*_0_, from which a *p*‐value was computed.

Tests contrasting the entire LD patterns of the AD‐affected and unaffected groups provide statistics for the association of the pattern of differences in LD between these two groups with ADs, called the molecular signature of ADs. The statistic for a given pair of groups does not require multiple testing correction. Significance of the *r* ^2^ estimates and the differences in the pairwise estimates of LD should be corrected for multiple testing. In the case of the 30 SNPs examined, this represented 435 (=30 × 29/2) tests. We adopted a conservative Bonferroni correction for significance, *p *<* *1.2 × 10^−4^, despite some correlation between these SNPs.

Asymptotically valid confidence intervals were constructed using asymptotic variance adapted from (Wellek & Ziegler, 2009). This asymptotic variance closely coincided with the exact variance in a sample of *n *≥* *60 individuals.

### 4.8 Functional annotation

Functional features and activity levels of the selected SNPs were annotated using the Ensembl variant effect predictor (McLaren et al., 2010) for 68 cell types. Information on expression quantitative trait loci was obtained from the GTEx pilot analysis, v6 (Consortium G, 2015). Chromatin state and protein binding annotation (Roadmap Epigenomics and ENCODE projects), and the effects of SNPs on regulatory motifs were annotated using HaploReg (Ward & Kellis, 2012) v.4.1 (http://archive.broadinstitute.org/mammals/haploreg/haploreg.php).

## AUTHOR'S CONTRIBUTION

A.M.K. conceived and designed the experiment and wrote the paper, J.H., J.W., L.H., and Y.L. prepared data, coded statistical tests, and performed statistical analyses. I.C. performed bioinformatics analysis.

## CONFLICT OF INTEREST

The authors declare that they have no conflict of interest.

## References

[acel12779-bib-0001] Artiga, M. J. , Bullido, M. J. , Sastre, I. , Recuero, M. , Garcıa, M. A. , Aldudo, J. , … Valdivieso, F. (1998). Allelic polymorphisms in the transcriptional regulatory region of apolipoprotein E gene. FEBS Letters, 421, 105–108. 10.1016/S0014-5793(97)01543-3 9468288

[acel12779-bib-0002] Charlesworth, B. (1996). Evolution of senescence: Alzheimer's disease and evolution. Current Biology, 6, 20–22. 10.1016/S0960-9822(02)00411-6 8805211

[acel12779-bib-0004] Corder, E. H. , Saunders, A. , Strittmatter, W. , Schmechel, D. , Gaskell, P. , Small, G. , … Pericak‐Vance, M. (1993). Gene dose of apolipoprotein E type 4 allele and the risk of Alzheimer's disease in late onset families. Science, 261, 921–923. 10.1126/science.8346443 8346443

[acel12779-bib-0005] Corella, D. , & Ordovas, J. M. (2014). Aging and cardiovascular diseases: The role of gene‐diet interactions. Ageing Research Reviews, 18, 53–73. 10.1016/j.arr.2014.08.002 25159268

[acel12779-bib-0006] Crespi, B. , Stead, P. , & Elliot, M. (2010). Evolution in health and medicine Sackler colloquium: Comparative genomics of autism and schizophrenia. Proceedings of the National Academy of Sciences of the United States of America, 107(Suppl 1), 1736–1741. 10.1073/pnas.0906080106 19955444 PMC2868282

[acel12779-bib-0007] Creyghton, M. P. , Cheng, A. W. , Welstead, G. G. , Kooistra, T. , Carey, B. W. , Steine, E. J. , … Jaenisch, R. (2010). Histone H3K27ac separates active from poised enhancers and predicts developmental state. Proceedings of the National Academy of Sciences of the United States of America, 107, 21931–21936. 10.1073/pnas.1016071107 21106759 PMC3003124

[acel12779-bib-0008] Cummings, J. L. , Morstorf, T. , & Zhong, K. (2014). Alzheimer's disease drug‐development pipeline: Few candidates, frequent failures. Alzheimers Research and Therapy, 6, 37 10.1186/alzrt269 PMC4095696 25024750

[acel12779-bib-0009] Cupples, L. A. , Heard‐Costa, N. , Lee, M. , Atwood, L. D. , Framingham Heart Study Investigators (2009) Genetics analysis workshop 16 problem 2: The Framingham Heart Study data. BMC Proceedings, 3(Suppl 7), S3 10.1186/1753-6561-3-s7-s3 PMC2795927 20018020

[acel12779-bib-0010] Deelen, J. , Beekman, M. , Uh, H. W. , Helmer, Q. , Kuningas, M. , Christiansen, L. , … Slagboom, P. E. (2011). Genome‐wide association study identifies a single major locus contributing to survival into old age; the APOE locus revisited. Aging Cell, 10, 686–698. 10.1111/j.1474-9726.2011.00705.x 21418511 PMC3193372

[acel12779-bib-0011] Finch, C. E. (2012). Evolution of the human lifespan, past, present, and future: Phases in the evolution of human life expectancy in relation to the inflammatory load. Proceedings of the American Philosophical Society, 156, 9–44. 23035388

[acel12779-bib-0012] Fitzsimons, C. P. , van Bodegraven, E. , Schouten, M. , Lardenoije, R. , Kompotis, K. , Kenis, G. , … Rutten, B. P. (2014). Epigenetic regulation of adult neural stem cells: Implications for Alzheimer's disease. Molecular Neurodegeneration, 9, 25 10.1186/1750-1326-9-25 24964731 PMC4080757

[acel12779-bib-0013] Fortney, K. , Dobriban, E. , Garagnani, P. , Pirazzini, C. , Monti, D. , Mari, D. , … Kim, S. K. (2015). Genome‐wide scan informed by age‐related disease identifies loci for exceptional human longevity. PLoS Genetics, 11, e1005728 10.1371/journal.pgen.1005728 26677855 PMC4683064

[acel12779-bib-0014] Fried, L. P. , Borhani, N. O. , Enright, P. , Furberg, C. D. , Gardin, J. M. , Kronmal, R. A. , … Weiler, P. G. (1991). The Cardiovascular Health Study: Design and rationale. Annals of Epidemiology, 1, 263–276. 10.1016/1047-2797(91)90005-W 1669507

[acel12779-bib-0015] Gerdes, L. U. (2003). The common polymorphism of apolipoprotein E: Geographical aspects and new pathophysiological relations. Clinical Chemistry and Laboratory Medicine, 41, 628–631. 12812258 10.1515/CCLM.2003.094

[acel12779-bib-0016] Goate, A. , Chartier‐Harlin, M. C. , Mullan, M. , Brown, J. , Crawford, F. , Fidani, L. , … Hardy, G. (1991). Segregation of a missense mutation in the amyloid precursor protein gene with familial Alzheimer's disease. Nature, 349, 704–706. 10.1038/349704a0 1671712

[acel12779-bib-0017] Grehan, S. , Tse, E. , & Taylor, J. M. (2001). Two distal downstream enhancers direct expression of the human apolipoprotein E gene to astrocytes in the brain. Journal of Neuroscience, 21, 812–822. 10.1523/JNEUROSCI.21-03-00812.2001 11157067 PMC6762321

[acel12779-bib-0003] GTEx Consortium . (2015). Human genomics. The Genotype‐Tissue Expression (GTEx) pilot analysis: Multitissue gene regulation in humans. Science, 348, 648–660. 25954001 10.1126/science.1262110 PMC4547484

[acel12779-bib-0018] Hardy, J. A. , & Higgins, G. A. (1992). Alzheimer's disease: The amyloid cascade hypothesis. Science, 256, 184–185. 10.1126/science.1566067 1566067

[acel12779-bib-0019] Harris, S. A. , & Harris, E. A. (2015). Herpes simplex virus type 1 and other pathogens are key causative factors in sporadic Alzheimer's disease. Journal of Alzheimer's Disease, 48, 319–353. 10.3233/JAD-142853 PMC4923765 26401998

[acel12779-bib-0020] Howie, B. N. , Donnelly, P. , & Marchini, J. (2009). A flexible and accurate genotype imputation method for the next generation of genome‐wide association studies. PLoS Genetics, 5, e1000529. 19543373 10.1371/journal.pgen.1000529 PMC2689936

[acel12779-bib-0021] Itzhaki, R. F. , Lathe, R. , Balin, B. J. , Ball, M. J. , Bearer, E. L. , Braak, H. , … Whittum‐Hudson, J. A. (2016). Microbes and Alzheimer's disease. Journal of Alzheimer's Disease, 51, 979–984. 10.3233/JAD-160152 PMC5457904 26967229

[acel12779-bib-0022] Jazwinski, S. M. , Kim, S. , Dai, J. , Li, L. , Bi, X. , Jiang, J. C. , … Georgia Centenarian Study and the Louisiana Healthy Aging Study (2010). HRAS1 and LASS1 with APOE are associated with human longevity and healthy aging. Aging Cell, 9, 698–708. 10.1111/j.1474-9726.2010.00600.x 20569235 PMC2941558

[acel12779-bib-0023] Juster, F. T. , & Suzman, R. (1995). An overview of the health and retirement study. Journal of Human Resources, 30, S7–S56. 10.2307/146277

[acel12779-bib-0024] Koistinaho, M. , Lin, S. , Wu, X. , Esterman, M. , Koger, D. , Hanson, J. , … Paul, S. M. (2004). Apolipoprotein E promotes astrocyte colocalization and degradation of deposited amyloid‐beta peptides. Nature Medicine, 10, 719–726. 10.1038/nm1058 15195085

[acel12779-bib-0025] Krzanowski, W. J. (1993). Permutational tests for correlation‐matrices. Statistics Computational, 3, 37–44.

[acel12779-bib-0026] Kulminski, A. M. (2013). Unraveling genetic origin of aging‐related traits: Evolving concepts. Rejuvenation Research, 16, 304–312. 10.1089/rej.2013.1441 23768105 PMC3746287

[acel12779-bib-0027] Lee, J. H. , Cheng, R. , Graff‐Radford, N. , Foroud, T. , Mayeux, R. , & National Institute on Aging Late‐Onset Alzheimer's Disease Family Study Group (2008). Analyses of the National Institute on Aging Late‐Onset Alzheimer's Disease Family Study: Implication of additional loci. Archives of Neurology, 65, 1518–1526. 10.1001/archneur.65.11.1518 19001172 PMC2694670

[acel12779-bib-0028] Lescai, F. , Chiamenti, A. M. , Codemo, A. , Pirazzini, C. , D'Agostino, G. , Ruaro, C. , … Franceschi, C. (2011). An APOE haplotype associated with decreased epsilon 4 expression increases the risk of late onset Alzheimer's disease. Journal of Alzheimer's Disease, 24, 235–245. 10.3233/JAD-2011-101764 21263195

[acel12779-bib-0029] Levy‐Lahad, E. , Wasco, W. , Poorkaj, P. , Romano, D. M. , Oshima, J. , Pettingell, W. H. , … Tanzi, R. E. (1995). Candidate gene for the chromosome 1 familial Alzheimer's disease locus. Science, 269, 973–977. 10.1126/science.7638622 7638622

[acel12779-bib-0030] Lewontin, R. C. (1988). On measures of gametic disequilibrium. Genetics, 120, 849–852. 3224810 10.1093/genetics/120.3.849 PMC1203562

[acel12779-bib-0031] Martin, E. R. , Gilbert, J. R. , Lai, E. H. , Riley, J. , Rogala, A. R. , Slotterbeck, B. D. , … Vance, J. M. (2000). Analysis of association at single nucleotide polymorphisms in the APOE region. Genomics, 63, 7–12. 10.1006/geno.1999.6057 10662539

[acel12779-bib-0032] McLaren, W. , Pritchard, B. , Rios, D. , Chen, Y. , Flicek, P. , & Cunningham, F. (2010). Deriving the consequences of genomic variants with the Ensembl API and SNP effect predictor. Bioinformatics, 26, 2069–2070. 10.1093/bioinformatics/btq330 20562413 PMC2916720

[acel12779-bib-0033] Murao, N. , Noguchi, H. , & Nakashima, K. (2016). Epigenetic regulation of neural stem cell property from embryo to adult. Neuroepigenetics, 5, 1–10. 10.1016/j.nepig.2016.01.001

[acel12779-bib-0034] Nesse, R. M. , Ganten, D. , Gregory, T. R. , & Omenn, G. S. (2012). Evolutionary molecular medicine. Journal of Molecular Medicine, 90, 509–522. 10.1007/s00109-012-0889-9 22544168 PMC4416654

[acel12779-bib-0035] Nielsen, D. M. , Ehm, M. G. , & Weir, B. S. (1998). Detecting marker‐disease association by testing for Hardy‐Weinberg disequilibrium at a marker locus. American Journal of Human Genetics, 63, 1531–1540. 10.1086/302114 9867708 PMC1377570

[acel12779-bib-0036] Oeppen, J. , & Vaupel, J. W. (2002). Demography. Broken limits to life expectancy. Science, 296, 1029–1031. 12004104 10.1126/science.1069675

[acel12779-bib-0037] Pekny, M. , Pekna, M. , Messing, A. , Steinhäuser, C. , Lee, J. M. , Parpura, V. , … Verkhratsky, A. (2016). Astrocytes: A central element in neurological diseases. Acta Neuropathologica, 131, 323–345. 10.1007/s00401-015-1513-1 26671410

[acel12779-bib-0038] Porcellini, E. , Carbone, I. , Ianni, M. , & Licastro, F. (2010). Alzheimer's disease gene signature says: Beware of brain viral infections. Immunity Ageing, 7, 16 10.1186/1742-4933-7-16 21156047 PMC3019140

[acel12779-bib-0039] Puri, D. , Gala, H. , Mishra, R. , & Dhawan, J. (2015). High‐wire act: The poised genome and cellular memory. FEBS Journal, 282, 1675–1691. 10.1111/febs.13165 25440020

[acel12779-bib-0040] Raichlen, D. A. , & Alexander, G. E. (2014). Exercise, APOE genotype, and the evolution of the human lifespan. Trends in Neurosciences, 37, 247–255. 10.1016/j.tins.2014.03.001 24690272 PMC4066890

[acel12779-bib-0041] Rogaev, E. I. , Sherrington, R. , Rogaeva, E. A. , Levesque, G. , Ikeda, M. , Liang, Y. , … St George‐Hyslop, P. H. (1995). Familial Alzheimer's disease in kindreds with missense mutations in a gene on chromosome 1 related to the Alzheimer's disease type 3 gene. Nature, 376, 775–778. 10.1038/376775a0 7651536

[acel12779-bib-0042] Roses, A. D. , Lutz, M. W. , Amrine‐Madsen, H. , Saunders, A. M. , Crenshaw, D. G. , Sundseth, S. S. , … Reiman, E. M. (2010). A TOMM40 variable‐length polymorphism predicts the age of late‐onset Alzheimer's disease. Pharmacogenomics Journal, 10, 375–384. 10.1038/tpj.2009.69 20029386 PMC2946560

[acel12779-bib-0043] Sherrington, R. , Rogaev, E. I. , Liang, Y. , Rogaeva, E. A. , Levesque, G. , Ikeda, M. , … St George‐Hyslop, P. H. (1995). Cloning of a gene bearing missense mutations in early‐onset familial Alzheimer's disease. Nature, 375, 754–760. 10.1038/375754a0 7596406

[acel12779-bib-0044] Sofroniew, M. V. (2015). Astrocyte barriers to neurotoxic inflammation. Nature Reviews Neuroscience, 16, 249–263. 10.1038/nrn3898 25891508 PMC5253239

[acel12779-bib-0045] Song, L. , Zhang, Z. , Grasfeder, L. L. , Boyle, A. P. , Giresi, P. G. , Lee, B. K. , … Furey, T. S. (2011). Open chromatin defined by DNaseI and FAIRE identifies regulatory elements that shape cell‐type identity. Genome Research, 21, 1757–1767. 10.1101/gr.121541.111 21750106 PMC3202292

[acel12779-bib-0046] Swerdlow, R. H. , Burns, J. M. , & Khan, S. M. (2014). The Alzheimer's disease mitochondrial cascade hypothesis: Progress and perspectives. Biochimica et Biophysica Acta, 1842, 1219–1231. 10.1016/j.bbadis.2013.09.010 24071439 PMC3962811

[acel12779-bib-0047] Vijg, J. , & Suh, Y. (2005). Genetics of longevity and aging. Annual Review of Medicine, 56, 193–212. 10.1146/annurev.med.56.082103.104617 15660509

[acel12779-bib-0048] Ward, A. , Crean, S. , Mercaldi, C. J. , Collins, J. M. , Boyd, D. , Cook, M. N. , & Arrighi, H. M. (2012). Prevalence of apolipoprotein E4 genotype and homozygotes (APOE e4/4) among patients diagnosed with Alzheimer's disease: A systematic review and meta‐analysis. Neuroepidemiology, 38, 1–17. 10.1159/000334607 22179327

[acel12779-bib-0049] Ward, L. D. , & Kellis, M. (2012). HaploReg: A resource for exploring chromatin states, conservation, and regulatory motif alterations within sets of genetically linked variants. Nucleic Acids Research, 40, D930–D934. 10.1093/nar/gkr917 22064851 PMC3245002

[acel12779-bib-0050] Weir, B. S. (1979). Inferences about linkage disequilibrium. Biometrics, 35, 235–254. 10.2307/2529947 497335

[acel12779-bib-0051] Weir, B. S. , & Cockerham, C. C. (1979). Estimation of linkage disequilibrium in randomly mating populations. Heredity, 42, 105–111. 10.1038/hdy.1979.10 4531429

[acel12779-bib-0052] Wellek, S. , & Ziegler, A. (2009). A genotype‐based approach to assessing the association between single nucleotide polymorphisms. Human Heredity, 67, 128–139. 10.1159/000179560 19077429

[acel12779-bib-0053] Willer, C. J. , Li, Y. , & Abecasis, G. R. (2010). METAL: Fast and efficient meta‐analysis of genomewide association scans. Bioinformatics, 26, 2190–2191. 10.1093/bioinformatics/btq340 20616382 PMC2922887

[acel12779-bib-0054] Zaykin, D. V. , Meng, Z. , & Ehm, M. G. (2006). Contrasting linkage‐disequilibrium patterns between cases and controls as a novel association‐mapping method. American Journal of Human Genetics, 78, 737–746. 10.1086/503710 16642430 PMC1474029

