# Supplementary material for: Apolipoprotein E region molecular signatures of Alzheimer's disease
Source: Aging Cell. 2018 May 23;17(4):e12779. doi: 10.1111/acel.12779 (PMC6052488; doi:10.1111/acel.12779)
Supplement: Supplementary file 1 [file ACEL-17-na-s001.docx]

**Figure S1. Forest plot illustrating association of rs157580 and rs2075650 (*TOMM40*) with AD.**


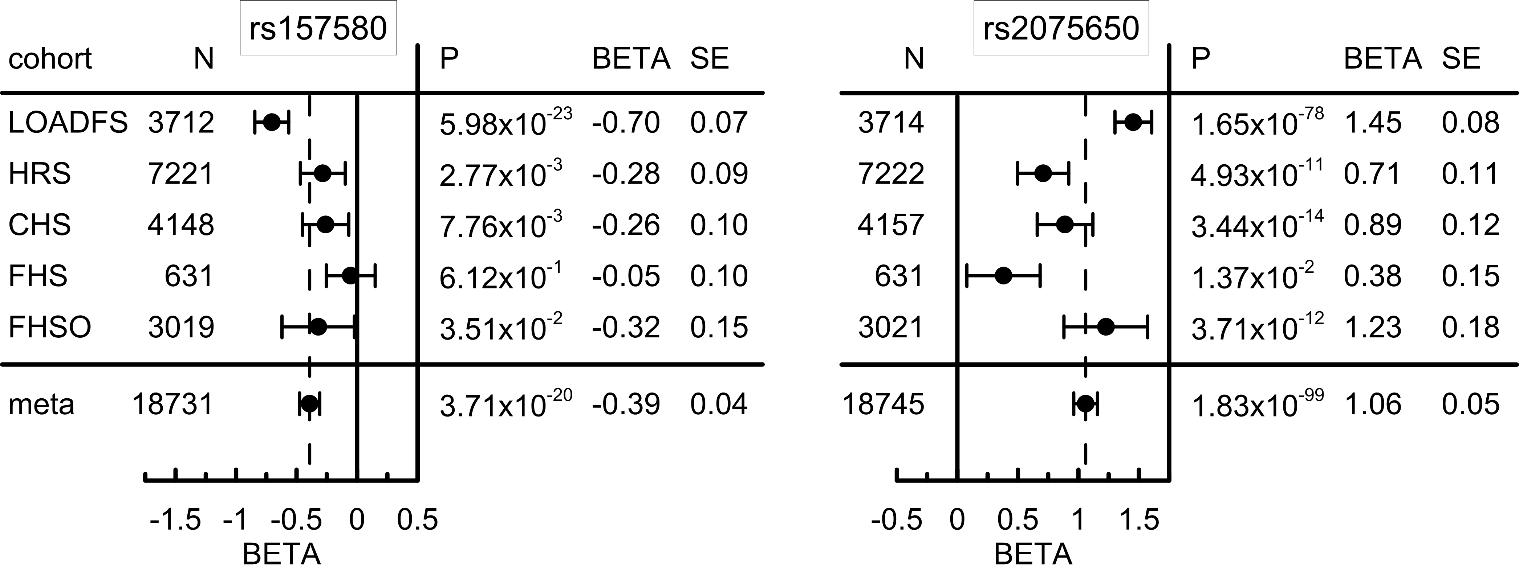


N denotes sample size in the pooled sample and in each cohort separately. Data are given in Table S2 (Supporting Information).
