# Supplementary material for: Apolipoprotein E region molecular signatures of Alzheimer's disease
Source: Aging Cell. 2018 May 23;17(4):e12779. doi: 10.1111/acel.12779 (PMC6052488; doi:10.1111/acel.12779)
Supplement: Supplementary file 2 [file ACEL-17-na-s002.docx]

**Figure S2. LD (*r^2^*, %) between SNPs from the *BCAM*-*NECTIN2* and *TOMM40-APOE-APOC1* loci.**


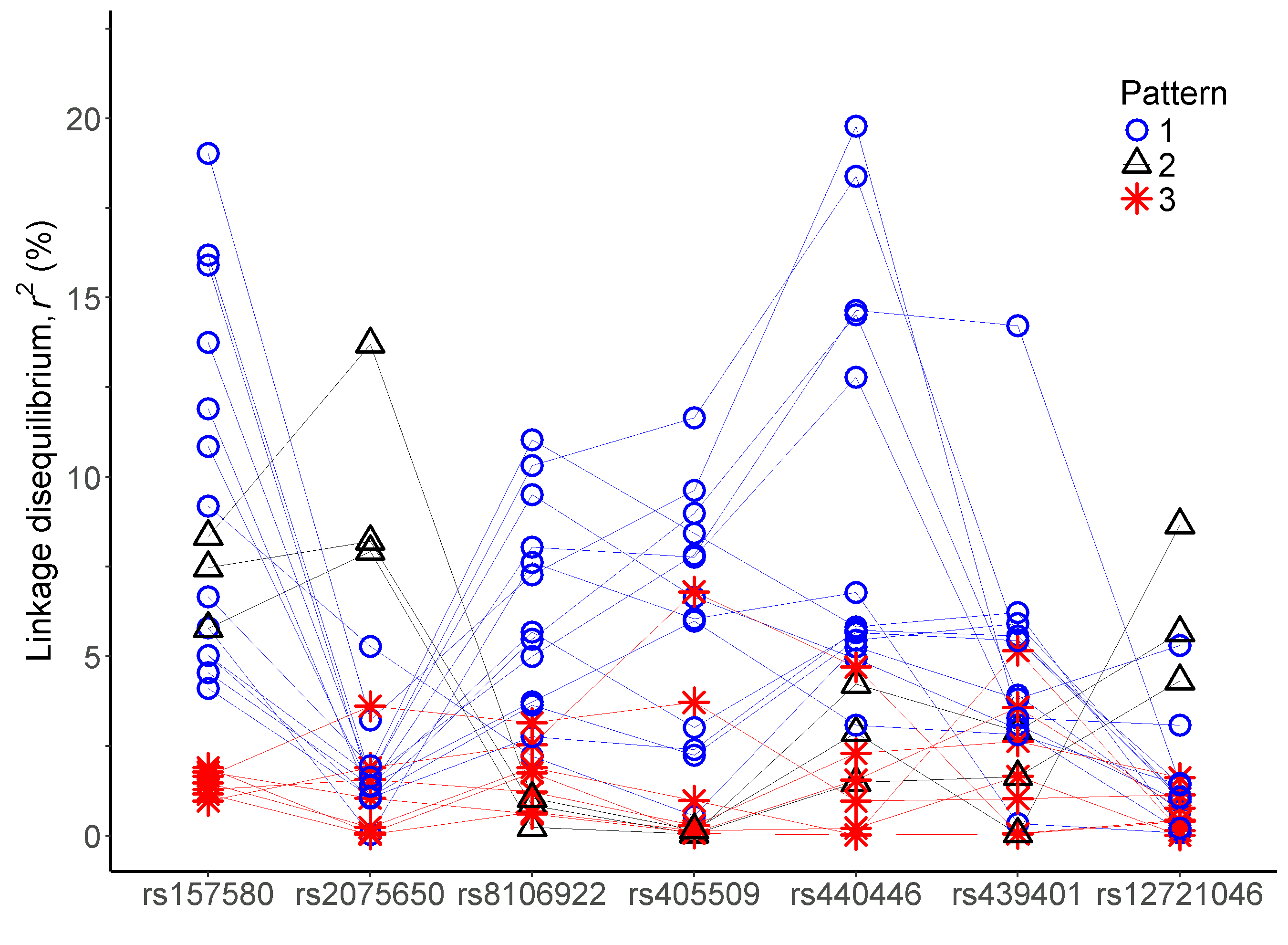


The structure is shown in the pooled sample of all cohorts for patterns 1, 2, and 3 (defined in Fig. 1).
