# Supplementary material for: Apolipoprotein E region molecular signatures of Alzheimer's disease
Source: Aging Cell. 2018 May 23;17(4):e12779. doi: 10.1111/acel.12779 (PMC6052488; doi:10.1111/acel.12779)
Supplement: Supplementary file 3 [file ACEL-17-na-s003.docx]

**Figure S3. Molecular signature of ADs defined by Δ*r^2^* and evaluated using the haplotype-based method.**


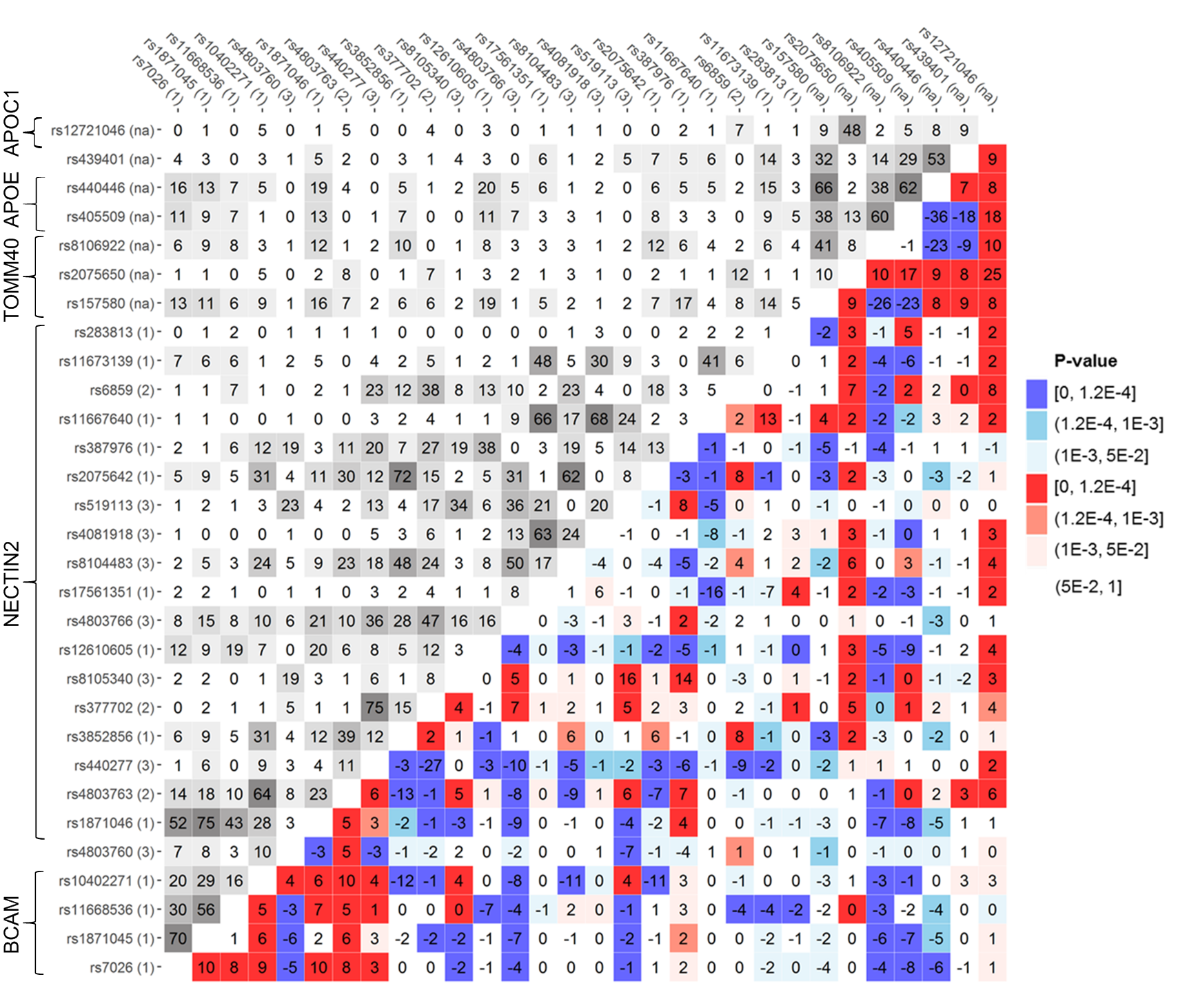


Upper-left triangle: LD pattern (*r^2^*, %) in the pooled sample of all studies, non-cases, for 30 SNPs. Lower-right triangle: heat map for Δ*r^2^*=*r^2^_cases_* – *r^2^_non-cases_* representing the molecular signature of ADs. Red denotes *r^2^_cases_* > *r^2^_non-cases_* and blue denotes *r^2^_cases_* < *r^2^_non-cases_*. Numbers 1-3 after SNP IDs indicate patterns shown in Fig. 1. Note that unbiased *p*-values are evaluated for Δ*r* because *r_cases_* and *r_n-c_* can be of opposite signs resulting in small Δ*r^2^* (e.g., Δ*r^2^*=0% but Δ*r* = -9% for rs6859 and rs439401). Legend on the right shows color coded p-values. Numerical estimates are given in Table S3 (Supporting Information).
