# Supplementary material for: Apolipoprotein E region molecular signatures of Alzheimer's disease
Source: Aging Cell. 2018 May 23;17(4):e12779. doi: 10.1111/acel.12779 (PMC6052488; doi:10.1111/acel.12779)
Supplement: Supplementary file 4 [file ACEL-17-na-s004.docx]

**Figure S4. Molecular signature of ADs defined by Δ*r* and evaluated using the genotype-based method.**
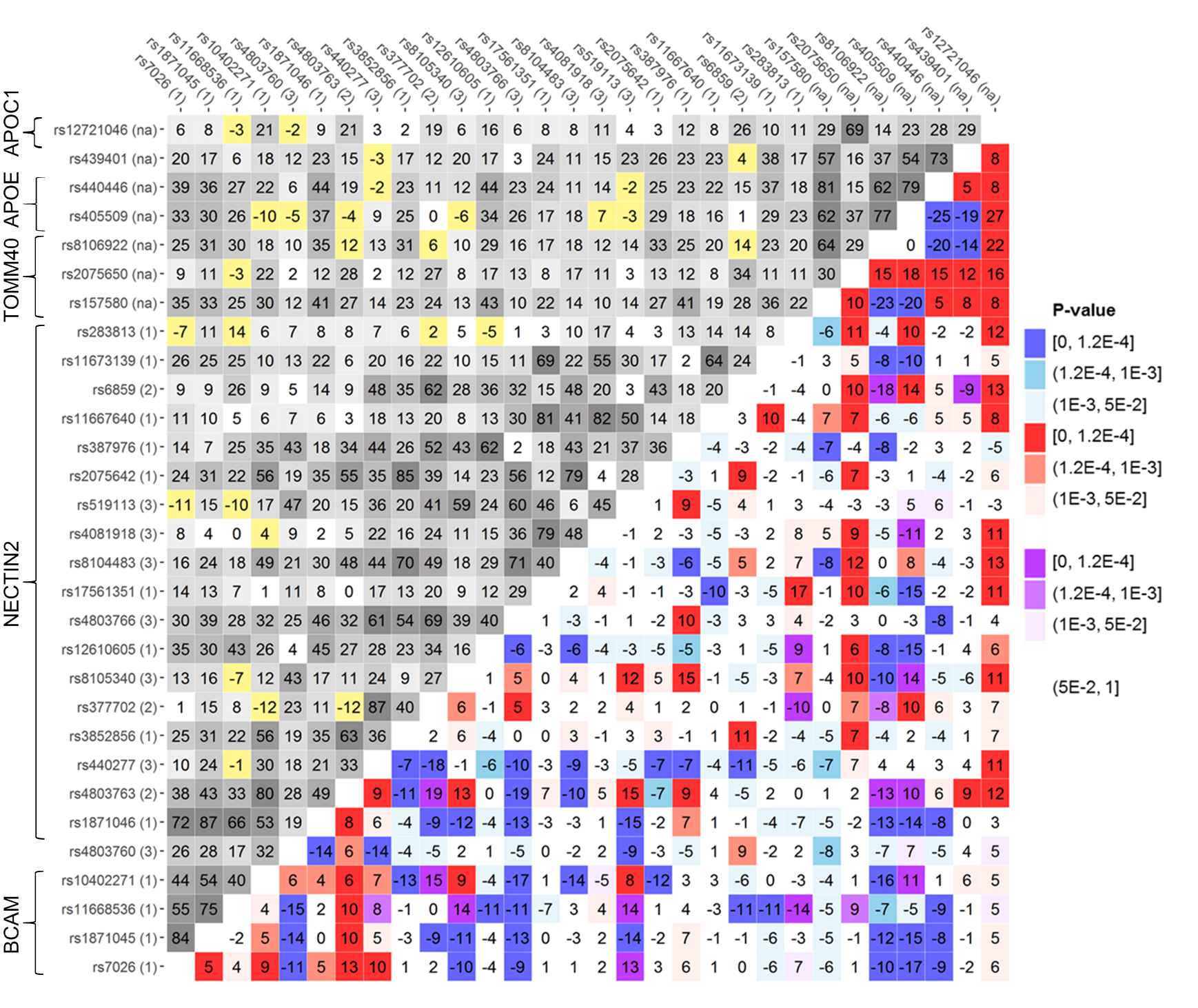


Upper-left triangle: LD pattern (*r*, %) in the pooled sample of all studies, non-cases, for 30 SNPs. Lower-right triangle: heat map for Δ*r* representing the molecular signature of AD. The difference Δ*r* was defined as Δ*r*=*r_cases_* - *r_non-cases_* if LD coefficients *r* were of opposite signs in cases and non-cases (yellow and purple), otherwise, Δ*r* was defined as Δ*r*=|*r_cases_*| - |*r_non-cases_*|. Red denotes *r_cases_* > *r_non-cases_* and blue denotes *r_cases_* < *r_non-cases_*. Numbers 1-3 after SNP IDs indicate patterns shown in Fig. 1. Legend on the right shows color coded p-values. We used *r* rather than *r^2^* here to emphasize that *r* can be of opposite signs in cases and non-cases. Numerical estimates for the pooled sample of all cohorts and for each cohort separately are shown in Table S4 (Supporting Information).
