# Supplementary material for: Apolipoprotein E region molecular signatures of Alzheimer's disease
Source: Aging Cell. 2018 May 23;17(4):e12779. doi: 10.1111/acel.12779 (PMC6052488; doi:10.1111/acel.12779)
Supplement: Supplementary file 5 [file ACEL-17-na-s005.docx]

**Figure S5. Molecular signatures of ADs in the *TOMM40-APOE-APOC1* locus for the directly genotyped SNPs.**


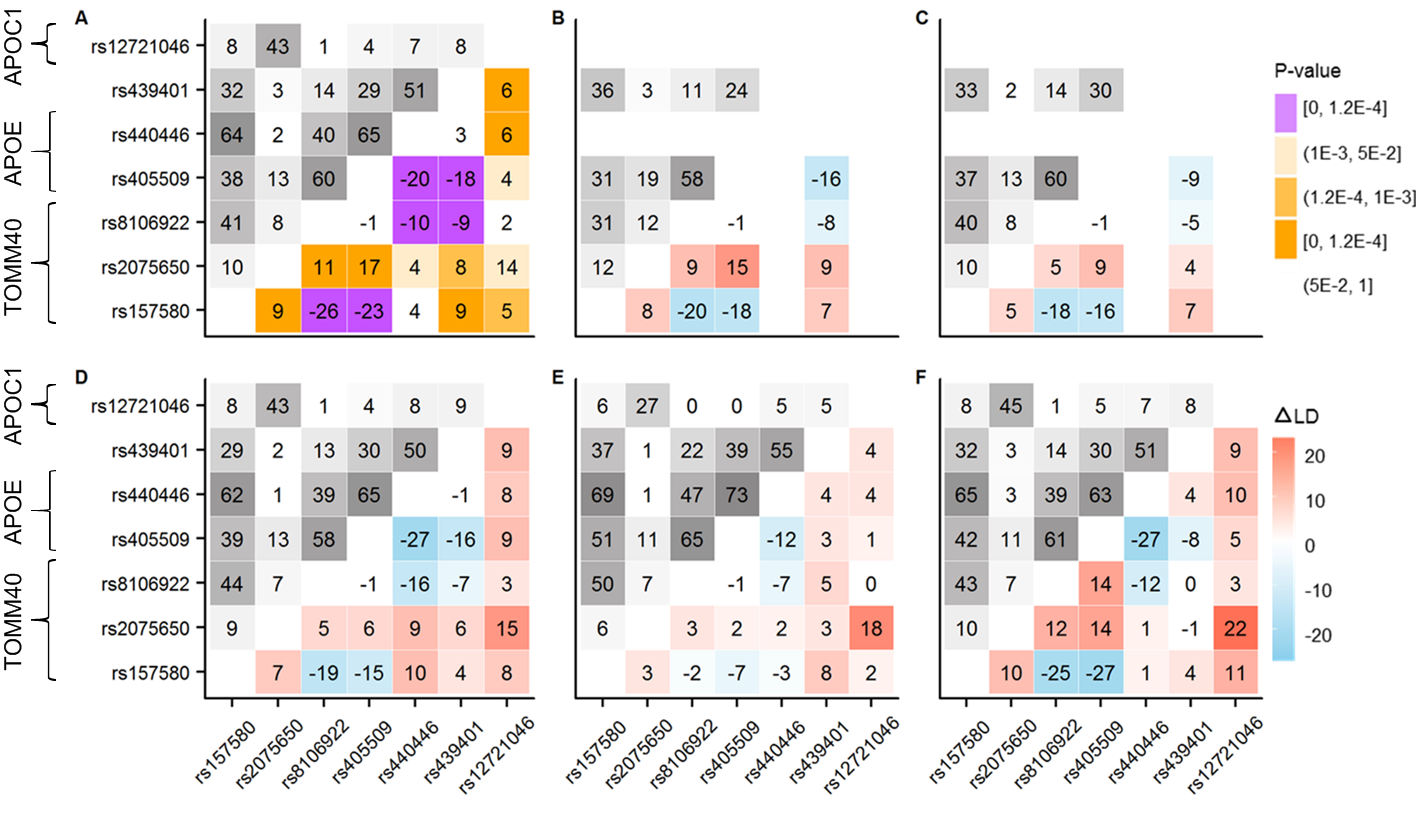


**(A)** Pooled sample of all cohorts, **(B)** Late Onset Alzheimer’s disease Family Study, **(C)** Health and Retirement Study, **(D)** Cardiovascular Health Study, **(E)** Framingham Heart Study (FHS) original cohort, and **(F)** FHS Offspring cohort. Upper-left triangle: LD pattern (*r^2^*, %) in non-cases for 7 SNPs representing the *TOMM40-APOE-APOC1* locus. Lower-right triangle: heat maps for Δ*r^2^*=*r^2^_cases_* – *r^2^_non-cases_* representing the molecular signature of ADs in this locus. Color in **(A)** codes p-values; color in **(B-F)** codes Δ*r^2^* (see legend). Only directly typed SNPs were included. White cells with no *r^2^* estimates denote not available SNPs.
