# Supplementary material for: Apolipoprotein E region molecular signatures of Alzheimer's disease
Source: Aging Cell. 2018 May 23;17(4):e12779. doi: 10.1111/acel.12779 (PMC6052488; doi:10.1111/acel.12779)
Supplement: Supplementary file 6 [file ACEL-17-na-s006.docx]

**Table S1. Basic demographic information for males and females genotyped in the selected studies.**

| Factor | LOADFS | | HRS | | CHS | | FHS | | FHSO | |
| --- | --- | --- | --- | --- | --- | --- | --- | --- | --- | --- |
|  | Non-cases | Cases | Non-cases | Cases | Non-cases | Cases | Non-cases | Cases | Non-cases | Cases |
| N | 1865 | 1850 | 6963 | 263 | 4074 | 252 | 426 | 205 | 2918 | 103 |
| Men (%) | 751 (40.3%) | 644 (34.8%) | 3030 (43.5%) | 99 (37.6%) | 1790 (43.9%) | 94 (37.3%) | 149 (35.0%) | 61 (29.8%) | 1332 (45.6%) | 51 (49.5%) |
| Birth year, mean (SD) | 1935.812 (11.5) | 1921.8 (8.4) | 1934.486 (8.3) | 1927.299 (7.1) | 1914.228 (5.7) | 1912.619 (5.7) | 1912.465 (3.9) | 1910.366 (4.4) | 1936.206 (9.4) | 1923.592 (6.9) |
| Age at baseline, mean (SD), years | 66.19 (11.5) | 80.93 (8.4) | 60.33 (8.7) | 67.66 (7.8) | 72.72 (5.6) | 74.29 (74.3) | 35.12 (4.0) | 37.11 (4.5) | 34.28 (9.5) | 47.02 (7.1) |
| Age at the end of follow up, mean (SD), years | 71.15 (10.7) | 83.53 (6.8) | 78.88  (8.0) | 85.16 (6.9) | 83.35 (5.4) | 85.15 (5.1) | 90.95 (4.8) | 92.21 (4.6) | 71.89 (9.1) | 82.02 (6.7) |
| Follow up through | 2015^*^ | 2015^*^ | 2012 | 2012 | 2002 | 2002 | 2012 | 2012 | 2012 | 2012 |

AD denotes Alzheimer's disease and related dementias.

N denotes genotyped sample after excluding individuals with missingness for SNPs greater than 5% and missing information on AD.

SD denotes standard deviation.

LOADFS = the NIA Late Onset Alzheimer’s disease Family Study; HRS = the Health and Retirement Study; CHS = the Cardiovascular Health Study; FHS = the Framingham Heart Study original cohort; FHSO = the FHS Offspring cohort.

Large proportion of AD cases in LOADFS is due to case-control design. Large proportion of AD cases in FHS is due to older age of this cohort at the end of follow up and larger proportion of women who are at higher risk of AD.

^*^ Information on age at onset of AD in LOADFS was not known for all cases.
